# Dietary antioxidant index and hypertension in the Iranian population: a nested case–control study within the Fasa adults cohort study

**DOI:** 10.3389/fnut.2024.1476122

**Published:** 2024-12-06

**Authors:** Mohammad amin Firooznia, Mehran Rahimlou, Eghbal Sekhavati, Mohammadreza Motazedian, Reza Tabrizi

**Affiliations:** ^1^Student research committee, Fasa University of Medical Sciences, Fasa, Iran; ^2^Department of Nutrition, School of Public Health, Zanjan University of Medical Sciences, Zanjan, Iran; ^3^Department of Occupational Health, Larestan University of Medical Sciences, Larestan, Iran; ^4^Clinical Research Development Unit, Valiasr Hospital, Fasa University of Medical Sciences, Fasa, Iran; ^5^Noncommunicable Diseases Research Center, Fasa University of Medical Sciences, Fasa, Iran; ^6^USERN Office, Fasa University of Medical Sciences, Fasa, Iran

**Keywords:** dietary antioxidant index, hypertension, oxidative stress, cardiovascular, diet

## Abstract

**Introduction:**

Hypertension (HTN) is a prevalent condition associated with numerous cardiovascular and non-cardiac complications. Lifestyle interventions, including dietary adjustments, offer promising avenues for hypertension management. However, the precise relationship between dietary antioxidants and hypertension risk necessitates further investigation. This study aimed to elucidate the association between the Dietary Antioxidant Index (DAI) and hypertension risk using a nested case–control design.

**Method:**

A matched nested case–control study was conducted within the Fasa Adult Cohort Study (FACS), comprising 975 participants aged 35–70 years. Cases (*n* = 325) were hypertensive patients, while controls (*n* = 650) were individuals without hypertension, matched for sex and age. Dietary intake was assessed using a validated food frequency questionnaire, and DAI was computed based on standardized intake of antioxidants. Conditional logistic regression models were employed to evaluate the association between DAI and hypertension risk, adjusting for confounding variables.

**Results:**

A significant inverse correlation was observed between DAI and hypertension risk across all models (OR = 0.89; 95% CI = 0.86–0.93, *p* < 0.001). This association remained robust after adjusting for potential confounders, including BMI, smoking, lipid profile, blood glucose levels, and educational status. In conclusion, higher DAI values were associated with a reduced risk of hypertension, highlighting the potential benefits of antioxidant-rich diets in hypertension prevention.

**Conclusion:**

These findings underscore the importance of dietary interventions as a complementary approach to hypertension management.

## Introduction

Hypertension (HTN), a condition that affects over 1 billion adults globally, is both a disease in itself and a significant contributing factor to other disorders (1, 2). The occurrence of hypertension increases dramatically around the world (3). HTN causes a substantial risk for a range of cardiovascular and several non-cardiac diseases (4, 5).

Treatment with antihypertensive medicines that lower blood pressure (BP) and related target organ damage can significantly minimize the heightened risk associated with BP elevation. However, despite the wide range of available treatment choices, nonadherence, intolerance, and resistant hypertension remain significant problems in pharmacological therapy (6). Antihypertensive medications are related to many side effects such as cough, edema, flushing, headache, increased urine output, rapid heart rate, wheezing, shortness of breath, and dizziness (7).

Conversely, there is increasing evidence that endorses the utilization of lifestyle strategies for both the prevention and complementary treatment of hypertension (8). Experimental evidence has shown that reactive oxygen species play a crucial role in the occurrence of hypertension (9). Therefore, adopting antioxidant-rich diets into lifestyle adjustments can be considered as a potential treatment option for high blood pressure (10). This process is explained by the imbalance between the production of reactive oxygen species and reactive nitrogen species, and the antioxidant defense systems, leading to oxidative and nitrosative stress in the cell. These mechanisms play a role in the arterial damage seen in chronic conditions like hypertension (11).

A previous study found that increasing the intake of fruits and vegetables in persons with hypertension for 6 months improved blood antioxidant capacity and lowered both systolic and diastolic blood pressure (12). Another study conducted by Waśkiewicz found a correlation between the total antioxidant capacity of the diet and polyphenol intake with a reduced risk of developing arterial hypertension in the Polish population (13).

However, there is a lack of comprehensive evaluations that thoroughly examine the complex connection between dietary antioxidant consumption and the risk of hypertension. This study aimed to clarify the association between the dietary antioxidant index (DAI) and hypertension through a nested case–control approach. The findings of this study could be offering valuable information for developing preventative measures and dietary guidelines for managing hypertension.

## Methods

### Study design and population

Information for this matched nested case–control study within the Fasa Adult Cohort Study (FACS) was collected from November 2014 to June 2019. The FACS is a comprehensive, population-based study conducted over an extended period. FACS included 10,035 individuals between the ages of 35 and 70 who did not have any physical or mental disabilities. These individuals were chosen randomly from the Sheshdeh rural area, with a total population of 41,000 people, and had been residents for a minimum of 9 months. This cohort study aimed to identify the risk variables linked to noncommunicable diseases in this particular population (14). This study was a matched nested case–control study. Cases were 325 patients that diagnosed with hypertension. Controls were 650 participants without hypertension. Controls were frequency-matched to cases by sex and age (±5 years), with a control: case ratio of 2:1. In the hypertensive group, hypertension stages were classified according to the standard guidelines from the American College of Cardiology (ACC) and American Heart Association (AHA), which define hypertension as follows: Stage 1 (systolic BP of 130–139 mmHg or diastolic BP of 80–89 mmHg) and Stage 2 (systolic BP ≥140 mmHg or diastolic BP ≥90 mmHg). Additionally, the hypertension group was assessed for common hypertension-related complications. Data on left ventricular hypertrophy (LVH) and kidney function status were recorded, and chronic kidney disease (CKD) was identified based on estimated glomerular filtration rate (eGFR) <60 mL/min/1.73 m^2^, where available. This additional information provides a more detailed clinical profile of the hypertensive participants and supports understanding of the overall cardiovascular risk in this group.

### Inclusion and exclusion criteria

The criteria for inclusion were as follows: a permanent residency in the area for a minimum of 2 years and willingness to participate in the study by providing informed consent through a signed agreement and to be in the age range of 20–80 years. Participants were not included in the analysis if they: suffering from autoimmune diseases, types of cancers, consumption of nutritional supplements with antioxidant properties such as vitamin E, selenium, vitamin A, zinc, vitamin C, omega 3, pregnancy and lactation, strict diet and gastrointestinal disorders such as irritable bowel syndrome (IBS), inflammatory bowel disease (IBD) and celiac. Also, people whose nutritional intake questionnaires were incompletely filled or whose caloric intake was abnormal (more than 4,500 kcal or less than 700 kcal) were excluded from the study.

### Assessment of dietary intake

The evaluation of patients’ dietary intake was conducted using a semi-quantitative food frequency questionnaire (FFQ) known for its validity and reliability, the questionnaire consisted of 168 popular food items consumed by Iranians, with standard serving sizes. The participants were asked about the frequency of consuming each food item, with responses categorized as daily, weekly, or monthly, and then converted to daily intakes. The gathered data underwent analysis through Nutritionist IV (First Databank, Hearst Corp., San Bruno, CA, USA). Participants who had not completed more than 10% of the dietary questionnaire items were eliminated from the study.

### Assessment of other variables

Enzymatic colorimetric method (Pars Azmoon, Tehran, Iran) was used to assess serum glucose, total cholesterol, triglyceride, and HDL cholesterol concentrations. Additionally, LDL cholesterol levels were determined using the Friedewald algorithm. Body weight was measured using a digital body mass scale and bioelectrical impedance with a precision of 0.01 kg using the Omron Karada Scan HBF-375 from Osaka, Japan. The BMI of each person was determined using the following formula: BMI = weight (in kg)/height (in m2).

Blood pressure was measured using the Omron HEM 7120 Automatic Blood Pressure (Omron, Japan). The measurement occurred after a resting interval of at least 10 min following recent activity. Participants were directed to sit comfortably with their left arm fully exposed and resting on a supported surface at heart level. Blood pressure measurements were taken on the left arm using the appropriate cuff size. Hypertension was described as having a systolic blood pressure (SBP) of 140 mmHg or higher and/or a diastolic blood pressure of 90 mmHg or higher, or taking blood pressure drugs regularly.

### Assessment of DAI

DAI for all study participants was determined using the data obtained from the FFQ. We employed databases that were published earlier and included the most frequently consumed foods to compute the DAI (15). For DAI computation, we standardized the intake of vitamin A, C, E, and selenium, manganese, and zinc by subtracting the global mean and dividing by the global standard deviation. The DAI was then determined by summing the standardized intakes, following the description provided below (16):


$$DAI=\sum_{i=1}^{N=6}\frac{\text{Individual intake}-\text{Mean}}{SD}\text{.}$$

Finally, to eliminate the effect of energy, the DAI score was adjusted to energy.

#### Statistical analysis

We utilized SPSS software version 16 for the data analysis. The continuous variables were presented as mean ± standard deviation, whereas categorical data were presented as frequency (percentage). To examine whether the variables are normally distributed, we used from the Kolmogorov–Smirnov test. In order to analyze the demographic variables, we used independent t test. Participants were categorized into quartiles according to their DAI score. Differences in general characteristics among dietary DAI quartiles and between cases and controls were evaluated using analysis of covariance (ANCOVA) for continuous variables and the Chi-square test for categorical variables. Lineaar regression was used to adjusted DAI score in term of total energy intake. Conditional logistic regression was employed in various models to explore the link between DAC and the risk of hypertension, with adjustments made for the most significant clinical variables. Initially, following control for BMI and smoking, the second model incorporated adjustments for educational levels and serum levels of TG, LDL, HDL and total cholestrol. In another model, supplementary adjustments involved FBS, SFA and PUFA. Confounding variables were chosen based on established relationships with both dietary antioxidant intake and hypertension risk, including BMI, smoking status, lipid profile, blood glucose levels, and educational status. These adjustments were made to control for potential confounding effects, although we acknowledge that certain variables, such as BMI and lipid profile, may act as mediators in the relationship between dietary antioxidants and hypertension.

Statistically significant values were defined as *p*-values less than 0.05.

## Results

In this matched case–control study, 975 participants (325 patients in case group and 650 people in control group) were included. The participants had a mean age of 51.24 ± 8.78 years Table 1 summarizes the demographic details of the study participants. The mean weight of the participants in the case group was 69.52 ± 13.95 kg and in the control group was 63.09 ± 12.68, and A statistically significant difference was found between the two groups (*p* < 0.001). Additionally, the mean systolic blood pressure (SBP) in the case group was 123.7 ± 18.52 mmHg, whereas it was 106.58 ± 15.24 mmHg in the control group, showing a significant disparity (*p* < 0.001). Furthermore, the diastolic blood pressure (DBP) level in the case group was 80.98 ± 12.43 mmHg, compared to 71.60 ± 10.97 mmHg in the control group, indicating a significant difference (*p* < 0.001). Regarding dietary intake, participants in the case group had a mean calorie consumption of 2737.52 ± 804.84 kcal/day, while the control group had a mean calorie intake of 2743.83 ± 860.78 kcal/day, with no significant differences (*p* = 0.91). Additionally, significant differences were observed between the case and control groups in terms of dietary vitamin E and zinc intake (*p* < 0.05). DAI value in hypertensive patients was −0.73 ± 4.18, whereas in the control group, it was 0.98 ± 4.48, indicating a diet with higher antioxidant content among controls (*p*-value <0.001). Table 2 showed differences in biochemical and nutritional variables among DAI quartiles. We found a significant differenes between DAI quartiles in term of age. Also, participants in the higher quartile of DAI compared to the subjects in the lower quartile consumed significantly higher amounts of enery, fat, carbohydrate, protein, sodium, satuarated fatty acid (SFA), mono and poly unsaturated fatty acid, dietary cholestrol, potassium, vitamin A, vitamin C, vitamin E, zinc, selenium and manganese (*p* < 0.001). However, we could not find any significant differences between DAI quartiles in term of lipid profile, blood sugar, SBP, DBP, weight and BMI (*p* > 0.05).

**Table 1:** General characteristics of the study population^a^.

Variables	Total (*n* = 975)	Case (*n* = 325)	Control (*n* = 650)	*P*_value^b^
DAI	0.41 ± 4.46	–0.73 ± 4.18	0.98 ± 4.48	<0.001
Age (y)	51.24 ± 8.78	51.24 ± 8.79	51.24 ± 8.78	1.00
Male (%)	288(29.5%)	96(29.5%)	192(29.5%)	1.00
Weight (kg)	65.23 ± 13.46	69.52 ± 13.95	63.09 ± 12.68	<0.001
BMI (kg/m^2^)	25.77 ± 4.92	27.28 ± 4.98	25.02 ± 4.71	<0.001
SBP (mmHg)	112.29 ± 18.28	123.7 ± 18.52	106.58 ± 15.24	<0.001
DBP (mmHg)	74.73 ± 12.29	80.98 ± 12.43	71.60 ± 10.97	<0.001
TG (mg/dl)	127.07 ± 70.39	144.94 ± 84.33	118.11 ± 60.34	<0.001
LDL (mg/dl)	112.37 ± 34.07	112.96 ± 34.64	112.07 ± 33.81	0.71
HDL (mg/dl)	50.37 ± 13.30	52.38 ± 15.90	49.36 ± 11.66	0.003
TC (mg/dl)	188.31 ± 40.32	194.82 ± 39.25	185.05 ± 40.49	<0.001
FBS (mg/dl)	94.08 ± 30.87	99.37 ± 39.28	91.36 ± 25.16	0.001
Smoking, no. (%)	234(25%)	85(26.15%)	149(23%)	0.38
Urban residence, *n* (%)	263 (27%)	80 (24.6%)	183 (28.2%)	0.23
Self-report diabetes, no, (%) (%)	146(14.97%)	71 (21.8%)	75 (11.5%)	<0.001
Education, no. (%)				0.38
High school or less	683(70%)	220(67.70%)	463(71.23%)	
College education (%)	292(30%)	105 (32.30%)	187 (28.77%)	
Dietary intake				
Energy, Kcal/day	2741.74 ± 842.14	2737.52 ± 804.84	2743.83 ± 860.78	0.91
Fat, g/day	68.24 ± 25.66	68.64 ± 26.05	68.03 ± 25.47	0.73
Carbohydrate, g/day	389.68 ± 93.37	390.96 ± 91.66	395.04 ± 94.28	0.76
Protein, g/day	86.25 ± 28.83	85.02 ± 26.44	86.86 ± 29.94	0.33
Cholesterol intake, mg/day	258.94 ± 146.97	252.12 ± 148.95	262.35 ± 145.97	0.31
Na (mg/day)	4389.44 ± 1428.41	4451.44 ± 1398.22	4358.48 ± 1443.32	0.34
SFA (g/day)	23.91 ± 10.33	85.57 ± 8.17	85.01 ± 8.00	0.55
MUFA (g/day)	18.29 ± 8.30	18.29 ± 8.35	18.30 ± 8.29	0.99
PUFA(g/day)	10.06 ± 67.37	9.86 ± 4.62	10.15 ± 5.36	0.663
Potassium (mg/day)	3811.88 ± 1569.89	3874.67 ± 1545.49	3780.54 ± 1582.19	0.38
Vitamin A (mcg/day)	744.11 ± 351.32	740.45 ± 364.46	745.01 ± 344.83	0.81
Vitamin C (mg/day)	141.38 ± 87.70	139.94 ± 83.54	142.11 ± 89.76	0.71
Vitamin E(mg/day)	7.84 ± 3.46	7.45 ± 3.38	8.04 ± 3.48	0.01
Zinc(mg/day)	10.76 ± 4.02	10.08 ± 3.97	11.11 ± 4.01	<0.001
Manganese(mg/day)	4.51 ± 2.53	4.21 ± 2.66	4.66 ± 2.44	0.41
Selenium(mcg/day)	132.14 ± 59.57	120.93 ± 49.21	137.75 ± 63.42	0.001

^a All data are means ± standard deviations unless indicated. b Calculated by independent t test. BMI, Body mass index; DBP, diastolic blood pressure; DAI, Dietary Antioxidant Inde; mg/dl, milligram/deciliter; FBS, Fasting blood sugar; HDL, high density lipoprotein; LDL, low density lipoprotein; Na, Sodium; SFA, saturated fatty acid; MUFA, mono unsaturated fatty acid; PUFA, poly unsaturated fatty acid; TG, triglyceride; TC, total cholesterol.^

**Table 2:** Distribution of characteristics and dietary intakes across tertiles of the DAI (*n* = 975) ^a^.

Variables	Q 1 (*n* = 243)	Q 2 (*n* = 244)	Q3 (*n* = 244)	Q 4 (*n* = 244)	*P*-value^b^
Age (y)	51.62 ± 8.72	52.24 ± 8.89	50.22 ± 8.70	50.90 ± 8.73	0.06
Weight (kg)	66.15 ± 14.37	65.53 ± 13.91	64.50 ± 12.67	64.75 ± 12.82	0.51
BMI (kg/m^2^)	26.23 ± 5.18	25.81 ± 4.86	25.53 ± 4.69	25.77 ± 4.92	0.35
SBP (mmHg)	112.87 ± 18.52	113.44 ± 19.10	111.68 ± 18.09	111.17 ± 17.37	0.22
DBP (mmHg)	74.70 ± 12.66	74.62 ± 12.54	74.93 ± 12.47	74.67 ± 11.55	0.32
TG (mg/dl)	133.27 ± 73.39	128.91 ± 75.30	124.07 ± 67.89	122.00 ± 64.30	0.29
LDL (mg/dl)	112.47 ± 37.46	117.11 ± 35.10	108.50 ± 29.64	111.36 ± 33.23	0.04
HDL (mg/dl)	49.70 ± 14.55	50.31 ± 12.81	50.63 ± 12.38	50.80 ± 13.48	0.59
TC (mg/dl)	189.06 ± 41.92	192.61 ± 40.79	184.20 ± 38.96	187.40 ± 39.35	0.12
FBS (mg/dl)	93.83 ± 33.36	92.08 ± 24.08	93.91 ± 29.93	96.29 ± 34.80	0.68
Energy, Kcal/day	2974.70 ± 873.12	2499.53 ± 739.22	2593.23 ± 831.05	2900.47 ± 828.07	<0.001
Fat, g/day	71.59 ± 24.29	60.60 ± 21.84	61.77 ± 22.47	79.24 ± 29.07	<0.001
Carbohydrate, g/day	400.12 ± 85.05	385.30 ± 97.45	366.07 ± 96.18	407.18 ± 89.51	<0.001
Protein, g/day	87.45 ± 29.29	78.92 ± 25.92	83.40 ± 28.18	95.32 ± 29.42	<0.001
Cholesterol intake, mg/day	218.90 ± 114.51	230.83 ± 124.38	250.64 ± 134.97	337.00 ± 177.79	<0.001
Na (mg/day)	4699.03 ± 1519.81	4047.25 ± 1280.16	4212.08 ± 1370.89	4601.21 ± 1438.33	<0.001
SFA (g/day)	25.24 ± 10.56	21.71 ± 9.22	21.77 ± 9.35	26.91 ± 11.16	<0.001
MUFA (g/day)	17.55 ± 7.77	16.24 ± 7.17	17.01 ± 6.84	22.45 ± 9.76	<0.001
PUFA (g/day)	8.29 ± 3.59	8.47 ± 3.50	9.73 ± 3.94	13.82 ± 6.74	<0.001
Potassium (mg/day)	3142.99 ± 1052.87	3207.59 ± 1020.54	3692.77 ± 1198.85	5222.62 ± 1871.33	<0.001
Vitamin A (mcg/day)	555.06 ± 259.59	615.52 ± 264.74	771 ± 316.85	1034.06 ± 345.90	<0.001
Vitamin C (mg/day)	86.41 ± 49.10	107.38 ± 53.50	138.78 ± 57.17	232.75 ± 98.63	<0.001
Vitamin E (mg/day)	5.90 ± 2.28	6.38 ± 2.22	7.88 ± 2.61	11.21 ± 3.70	<0.001
Zinc (mg/day)	10.13 ± 3.62	9.39 ± 3.31	10.56 ± 3.78	12.98 ± 4.39	<0.001
Manganese (mg/day)	4.09 ± 2.21	3.95 ± 1.93	4.32 ± 2.30	5.67 ± 3.13	<0.001
Selenium (mcg/day)	134.80 ± 51.16	118.98 ± 49.90	127.95 ± 57.89	146.85 ± 73.25	<0.001

^a All data are means ± standard deviations unless indicated. b One-way ANOVA was used for continuous variables. BMI, Body mass index; DBP, diastolic blood pressure; DAI, Dietary Antioxidant Inde; mg/dl, milligram/deciliter; FBS, Fasting blood sugar; HDL, high density lipoprotein; LDL, low density lipoprotein; Na, Sodium; SFA, saturated fatty acid; MUFA, mono unsaturated fatty acid; PUFA, poly unsaturated fatty acid; TG, triglyceride; TC, total cholesterol.^

ORs and 95% CIs for the odds of hypertension according to the DAI value are shown in Table 3. It has been reported that there was a significant inverse correlation between DAI and odds of hypertension in the crude model (OR = 0.91;95%CI = 0.88–0.94, *p* < 0.001). Also, in all of the adjusted models and after adjustment for confounding factors including BMI, smoking, LDL, FBS, HDL, TG, total cholestrol, educational degree, SFA and PUFA, a reverse and significant association were seen (OR = 0.89;95%CI = 0.86–0.93, *p* < 0.001).

**Table 3:** Multivariate-adjusted ORs and 95% CIs for hypertension in relation to dietary antioxidant index.

	OR (CI)	*P*
Crude	0.91 (0.88, 0.94)	<0.001
Model 1	0.92(0.88,0.944)	<0.001
Model 2	0.9(0.87,0.93)	<0.001
Model 3	0.89(0.86,0.93)	<0.001

Model 1: Adjusted for BMI, smoking. Model 2: Further adjusted for educational degree, LDL, HDL, cholesterol. Model 3: Further adjusted for FBS, SFA and PUFA.

## Discussion

This study aimed to investigate the potential relationship among DAI and hypertension. The importance of investigating this connection resides in the wider framework of public health, due to the increasing incidence of hypertension and the potential influence of dietary determinants on its onset. The study’s findings revealed fascinating patterns that warrant additional discussion, elucidating the complex interaction between dietary habits and hypertension incidence.

The observed odds ratio (OR = 0.89) indicates that each unit increase in DAI is associated with an 11% reduction in hypertension risk. In our study, DAI range was wide. This suggests that individuals moving from a lower to a higher DAI category could potentially reduce their hypertension risk by 11%. Translating these findings into practical dietary changes, an increase in DAI can be achieved through a higher intake of antioxidant-rich foods, including fruits, vegetables, whole grains, and legumes, which are known to contribute to oxidative stress reduction and blood pressure management. For instance, increasing daily servings of vegetables and fruits by five servings could significantly increase DAI, aligning with observed reductions in hypertension risk. Such dietary adjustments not only promote general cardiovascular health but may also serve as accessible strategies for hypertension prevention, with particular relevance for individuals at higher risk of hypertension.

The results indicated that in all models of multiple adjusted ORs, there was a significant and negative correlation between hypertension and the DAI. This finding aligns with the outcomes of prior research. In a cross-sectional investigation, Fateh et al. (17) examined the correlation between greater total antioxidant capacity in the diet and a lower risk of hypertension in pre/perimenopausal women. Their modeling using logistic regression analysis revealed a decreased hypertension risk in women with elevated dietary total antioxidant capacity (17). Moreover, the study conducted by Peng et al. (18) suggested a negative link between dietary antioxidants and hypertension risk among the Chinese middle-aged and older adults (18, 19). Another research has identified a potential link between diet and blood pressure in Iranian Kurdish women indicates that consuming a diet filled with antioxidants may be linked to a reduced likelihood to develop hypertension. (20). Similar results were seen in French women (21). Moreover, data from the National Health and Nutrition Examination Survey (NHANES) showed a potential link between higher intakes of dietary carotenoids, a type of antioxidant, and lower prevalence of hypertension in American adults (22).

The inverse association between DAI and hypertension risk observed in this study may be partly explained by several biological mechanisms through which antioxidants affect blood pressure regulation. Antioxidants play a crucial role in neutralizing reactive oxygen species (ROS), thereby reducing oxidative stress, which is known to contribute to vascular damage and hypertension (23). High levels of oxidative stress can impair endothelial function by decreasing the availability of nitric oxide (NO), a critical molecule that promotes vasodilation and regulates blood pressure. By reducing oxidative stress, dietary antioxidants may enhance endothelial function and improve NO bioavailability, thus aiding in blood pressure control. Additionally, dietary antioxidants have been associated with anti-inflammatory effects, which may also contribute to blood pressure regulation (24, 25). Chronic low-grade inflammation is increasingly recognized as a factor in hypertension pathogenesis. Antioxidant nutrients, such as vitamins C and E and polyphenols, have been shown to decrease inflammatory markers like C-reactive protein (CRP) and interleukin-6 (IL-6), which are elevated in hypertensive individuals. By mitigating inflammation, antioxidants may further reduce the risk of hypertension development (26, 27).

When free radicals accumulate in cells and tissues, exceeding the body’s natural antioxidant defenses, an imbalance called “oxidative stress” occurs (28). This stress damages cells and contributes to the development of CVD (29). Fortunately, antioxidants can counteract this process by donating electrons to free radicals, neutralizing their harmful potential and shielding the organism from harmful consequences of oxidative stress (30). Some researchers have suggested that part of the beneficial effects of diets with a high DAI on blood pressure is due to the high consumption of vitamin E and manganese in these diets (31). Research suggests a complex relationship between specific nutrients and hypertension. While vitamins E and manganese showed promise in some studies, findings were mixed across populations. Vitamin E intake exhibited a reverse J-shaped association with hypertension in Chinese adults (cohort study) (32), but no link was found in a Spanish study (33). This highlights the potential influence of ethnicity on such interactions. Conversely, meta-analyses consistently support the benefit of magnesium supplementation in lowering blood pressure (34–36).

Extensive research suggests that consuming foods rich in antioxidants might lower the likelihood of developing hypertension. The DASH (Dietary Approaches to Stop Hypertension) diet, which emphasizes fruits, vegetables, low-fat dairy, and whole grains, has consistently been proven to lower blood pressure in both healthy and hypertensive patients (37). This diet has also been linked to a decrease in cardiovascular complications like stroke, which are often associated with hypertension (38). While several studies suggest a link between antioxidant-rich diets and lower blood pressure risk, findings on supplementing with antioxidants are less conclusive, particularly when considering gender differences. In the Linxian trial, for example, men receiving antioxidant supplements had a decreased risk of developing hypertension after 6 years, while no such benefit was observed in women (39). While some studies suggest potential benefits of antioxidant supplements for blood pressure, others, like the SUVIMAX trial, have found no significant association with preventing hypertension (40). This raises the question of whether the naturally balanced mix of antioxidants found in whole foods might be more effective than isolated supplements, which could potentially disrupt the body’s delicate antioxidant system by providing an excess of specific antioxidants.

Multiple studies across humans and animal models show clear differences between genders or sexes in both how often high blood pressure occurs and how the body regulates it (41). Existing evidence suggests a sex-based disparity in Male spontaneous hypertension rat (SHR), with males exhibiting higher oxidative stress levels contributing to their hypertensive state compared to females. Consequently, this implies a potential for greater blood pressure reduction in males upon antioxidant administration compared to females (42). Although prior research suggests a gender-dependent relationship between the variables, our findings revealed a consistently inverse correlation regardless of participant gender.

The observed phenomenon might share a close link with oxidative stress. Existing literature (43, 44) indicates elevated oxidative stress and inflammation in hypertensive individuals. Manganese and zinc are essential elements in antioxidant mitochondrial metalloenzymes (MnSOD, ZnSOD) (45), Selenium, present in selenoproteins, helps prevent lipid peroxidation and oxidative cellular damage. This indicates a possible function for these micronutrients in lessening the observed effects (46). The crucial role of non-enzymatic antioxidants like vitamins A, C, and E in counteracting stress-induced oxidant alterations is emphasized in previous studies. This underscores the potential application of dietary antioxidant sources for the prevention of hypertension triggered by oxidative stress (31, 47).

In this study, we observed notably lower blood pressure values in the control group, which could reflect specific characteristics of the Iranian rural population sampled in the Fasa Adult Cohort Study (FACS). Several factors may account for these findings. First, rural populations in Iran, including the FACS cohort, often engage in traditional lifestyles that include higher physical activity levels and diets rich in fruits, vegetables, and whole grains. These foods contribute to higher DAI values, which are known to mitigate oxidative stress, a factor closely linked to hypertension development (48). Additionally, dietary patterns in rural Iranian settings may provide a variety of antioxidants naturally present in local foods, potentially explaining the observed lower baseline blood pressure in controls. The relationship between antioxidants and blood pressure has been documented, and diets rich in these compounds may support vascular health and reduce hypertension risk (49). Furthermore, unique genetic and environmental interactions specific to this region may also play a role in maintaining lower blood pressure. Previous studies have suggested that Iranian populations may exhibit distinct genetic profiles that, when combined with rural environmental influences, contribute to lower baseline blood pressure values (50).

The present study employs a nested case–control design within a large cohort study, allowing for a robust evaluation of the association between DAI and hypertension. This design helps control for potential confounding factors and provides valuable insights into the relationship being studied.

### Limitations

This study has several limitations. First, dietary intake was assessed using a validated FFQ, which is prone to recall bias and measurement error. Although the FFQ provides valuable insights into dietary patterns, its reliance on self-reported data may have affected the accuracy of the DAI calculation. Second, some variables included as confounders in the analysis, such as BMI and lipid profile, may act as mediators rather than confounders, potentially leading to over-adjustment and attenuation of the observed association. Third, the absence of objective biomarkers of antioxidant intake limits the ability to validate self-reported dietary data, which future studies should address. Finally, the generalizability of our findings may be restricted by the specific rural population studied, as dietary habits and genetic factors may differ across populations. Despite these limitations, the study provides valuable evidence on the relationship between dietary antioxidants and hypertension risk.

## Conclusion

In conclusion, our study offers supportive evidence for a significant inverse association between the DAI and hypertension risk, highlighting the potential benefits of antioxidant-rich diets in managing hypertension. Future studies employing more precise dietary assessment methods and interventional designs are warranted to confirm these associations and strengthen the basis for dietary recommendations in hypertension prevention.

## Data Availability

The raw data supporting the conclusions of this article will be made available by the authors, without undue reservation.
